# Structural insights of a PI3K/mTOR dual inhibitor with the morpholino-triazine scaffold

**DOI:** 10.1007/s10822-016-9905-4

**Published:** 2016-03-08

**Authors:** Takako Takeda, Yanli Wang, Stephen H. Bryant

**Affiliations:** National Center for Biotechnology Information, National Library of Medicine, National Institutes of Health, Bethesda, MD 20894 USA

**Keywords:** PI3K, mTOR, Pharmacophore, 3D-QSAR, Docking, PKI-587

## Abstract

Stimulation of the PI3K/Akt/mTOR pathway, which controls cell proliferation and growth, is often observed in cancer cell. Inhibiting both PI3K and mTOR in this pathway can switch off Akt activation and hence, plays a powerful role for modulating this pathway. PKI-587, a drug containing the structure of morpholino-triazines, shows a dual and nano-molar inhibition activity and is currently in clinical trial. To provide an insight into the mechanism of this dual inhibition, pharmacophore and QSAR models were developed in this work using compounds based on the morpholino-triazines scaffold, followed by a docking study. Pharmacophore model suggested the mechanism of the inhibition of PI3Kα and mTOR by the compounds were mostly the same, which was supported by the docking study showing similar docking modes. The analysis also suggested the importance of the flat plane shape of the ligands, the space surrounding the ligands in the binding pocket, and the slight difference in the shape of the binding sites between PI3Kα and mTOR.

**Electronic supplementary material:**

The online version of this article (doi:10.1007/s10822-016-9905-4) contains supplementary material, which is available to authorized users.

## Introduction

The phosphatidylinositol-4,5-bisphosphate 3-kinase (PI3K)/serine/threonine-specific protein kinase (Akt)/mammalian target of rapamycin (mTOR) pathway regulates cell proliferation and cell growth and is often stimulated in cancer, which makes it an important target pathway for cancer therapies [1, 2]. Activation of Akt is responsible for cell proliferations and cell translation. Akt is activated by PI3K indirectly, which results in the phosphorylation at Thr 308, while mTORC2 (mTOR complex 2) can activate Akt by phosphorylating Ser 473. mTORC1 (mTOR complex1) is downstream of Akt and can produce a negative feedback on the PI3K signaling activation. To fully activate Akt, phosphorylation of both Thr 308 and Ser 473 is necessary. Interestingly, although the sequence identity of the catalytic sites was low (Supplementary Material), PI3K and mTOR share a high structure similarity at their catalytic sites. Therefore, a drug with dual inhibition activity for both PI3K and mTOR may be developed to shut down Akt activation.

Dual inhibitors of PI3K/mTOR with various scaffolds have been developed. Some of these inhibitors are in clinical trials including BGT226 [3], NVP-BEZ235 [4], XL765 [5] and PKI-587 [6, 7]. PKI-587 has been designed based on the scaffold of morpholino-triazines, shows a sub-nano molar potency, and has attracted many research interests with recent studies demonstrating multiple clinical advantages. Recently it has been reported that PKI-587 can help cetuximaub (an inhibitor of epidermal growth factor receptor) to increase its sensitivity in resistant cell lines [8]. Also, PKI-587 inhibits the propagation of the cancer stem cell in liver with and without sorafenib [9] although the mechanism of action for this bioactivity is unclear. Clinical information about PKI-587 can be found in the clinical trials database (ClinicalTrials.gov) with multiple data entries: NCT02438761, phase II, for assessment of its “efficacy for patients with myeloid neoplasm secondary to chemo-radiotherapy (t-AML/MDS) and refractory AML”; and NCT01920061, phase I, for “assessment of its safety and tolerability in combination with other anti-tumor agents (Docetaxel, Cisplatin, Dacomitinib)” [10–12].

Pharmacophore modeling, 3D-QSAR (quantitative structure activity relationship) modeling, and docking are widely used in computer-aided drug design approaches. Pharmacophore modeling identifies the common structural and physicochemical features of a set of compounds that bind to the target molecules. QSAR modeling constructs mathematical formula between molecular structure features and its biological activities so that it can be used for screening chemical database for new lead compounds [13]. Docking studies can predict the binding mode and provide insight into the interaction between the ligand and the target.

The study of the structure and bioactivity relationships using the scaffold that led to the development of PKI-587 may provide molecular insights to the inhibition activity of this dual inhibitor and facilitate further development of additional dual PI3K/mTOR inhibitors and drugs. The aims of this study were to investigate the molecular basis of the inhibition against PI3K/mTOR and to identify the structure features of the compounds with morpholino-triazine scaffold that primarily contribute to the inhibition of PI3K/mTOR. We have conducted pharmacophore modeling, atom-based QSAR, and molecular docking studies, which consistently showed that the mechanisms for inhibiting PI3K and mTOR were mostly the same. The docking study showed that the compounds formed hydrogen bonds (HBs) with the corresponding residues that form HBs with ATPs in the X-ray crystallography structures of PI3Kγ. In addition, the outmost active compounds formed a HB with the amine moiety on the other end of the molecule, which showed as the main difference between the most active and the least active compound in the docking study. Similarity of the binding modes of PKI-587 to PI3K and mTOR suggested it is important to the dual inhibitor design. Docked complex structures for the most active compounds were compared to the selective/multi-target inhibitors’ complex structures with the enzymes.

## Methods

### Compounds and their activities

Activities (IC_50_) of bis (morpholino-1,3,5-triazine) derivatives for PI3Kα and mTOR were retrieved from PubChem Assay [14] (PI3Kα—AID 460017, AID 609982, and mTOR—AID 460019, AID 610010) based on two articles [6, 7] and a total 40 compounds are shown with PubChem compound ID (CID) [15] in Table S1 in Supplementary Material. 2D-molecular structures for these compounds were downloaded from PubChem as 2D-SDF files. As mutations and overexpression of PI3KCA, the gene of PI3Kα, are observed in many cancers, PI3Kα was chosen for the modeling among PI3K isoforms.

### Pharmacophore and QSAR modeling

The Phase program on Maestro (Schrödinger, LLC, New York, NY, 2013) was used for pharmacophore modeling and 3D-QSAR modeling. IC_50_ values were converted to pIC_50_. 2D-structures were converted to 3D structures and energy minimization was performed. The conformers were generated by ConfGen [16] equipped in Phase with the default parameters: the number of conformers per rotatable bond was set to 100, the maximum number of conformers per structure was set to 1000 with distance-dependent dielectric solvation model, and energy minimization was performed using the OPLS2005 force field. Pharmacophore models were built with the five most active compounds (MAC) for each enzyme. The least active compounds were used to deny hypotheses that do not distinguish between most active and least active compounds. These compounds were shown in Table S3 in Supplementary Material. The number of pharmacophore sites and minimum matches was set to five. The common pharmacophore search was carried out by binary decision tree using the distances between two sites. For scoring parameters, the weight of selectivity score was set to 1.0 and the other parameters were kept as default. High selectivity scores indicate the hypothesis is unique to the actives.

The compounds in the training set aligned to the top pharmacophore hypotheses were used for building atom-based QSAR model by partial least square (PLS) regression [17] with the maximum factor set to 3. The PLS method builds a set of models with different number of factors. Larger numbers of factors lead to better description of the training set at the risk of overfitting. In this study, we used the PLS method to build three sets of models (i.e. maximum factor = 3), and we chose the most accurate but not over-fitted model. In atom-based QSAR, atoms were classified into six types: hydrogen-bond donor (D), hydrophobic or nonpolar (H), negative ionic (N), positive ionic (P), electron-withdrawing (W), and others (X). To represent an atom, a spherical model with van der Waals radius of its atom types was used. A rectangular grid was set to cover the space of the aligned training set compounds and divided by 1 Å^3^ cubes. The independent variables of PLS regression take values of 0 or 1 depending on the cubes and its occupied atom types. The activity of the compounds was the dependent variable. Training set and test set for the QSAR models were selected so that the activities of the compounds were uniformly distributed for both sets with the ratio of the training set size over the test set size as 1:1. Two compounds (CID56683918 and 56683919) were removed for QSAR building because they were not aligned well to the pharmacophore hypothesis. For QSAR building of mTOR, one compound (CID56683917) was removed as an outlier.

### Docking

We docked PKI-587, CID 53379513 (MAC, the most active compound), and CID 46228569 (LAC, the least active compound) to PI3Kα. We also docked PKI-587, CID 44514208(MAC), and CID 56683919(LAC) to mTOR. Induced Fit Docking (IFD) was carried out, which used Glide version 6.1 and Prime version 3.4 (Schrödinger, LLC, New York, NY, 2013) [18, 19] on Maestro version 9.6. The IFD protocol was employed and the default settings were chosen except the trim side chain option was selected.

The protein structures of PI3Kα and mTOR were taken from the Protein Data Bank (PDB) with PDB ID code 4L23 and 4JT6 [20], respectively. Both protein structure complexes contain the same ligand in the PDB files. The Protein Preparation module on Maestro with default settings was used to prepare the protein structures.

2D-SDF files for small molecule compounds downloaded from PubChem were prepared by LigPrep, version 2.8 (Schrödinger, LLC, New York, NY, 2013) with default settings with OPLS2005 force field. Box size was set to “Dock ligands with length ≤50 Å” for MAC and to “Auto” for LAC since MAC were larger than LAC and the ligand in the original PDB files. The Induced Fit program reproduced the complex in 4JT6 with ligand-RMSD 0.85 ± 0.43 Å for the top 5 ranked structures from docked complex outputs. 98 % of the residues located within 4 Å from the ligand in the original complex structure remained close (within 4 Å) to the ligand in the top 5 ranked structures from the docked complexes. MAC for PI3Kα and that for mTOR were larger than the ligand in 4L23. Hence, for testing docking with a ligand with comparable size, we used 3IBE, a structure complex of PI3Kγ that has a larger ligand. Similarly to the docking result for 4JT6, the docking result for 3IBE through induced fit docking reproduced the structure complex with an average ligand-RMSD 1.50 ± 0.98 Å for the top 5 ranked structures. 95 % of the residues within 4 Å to the ligand in the original complexes remained close (within 4 Å) to the ligand in the top 5 ranked docked complexes.

Induced fit docking was carried out as flexible conformations can be generated depending on the various size of the compounds. It allows protein side chain and backbone movements. This is necessary since the compound sizes in this study were larger than that of the ligands in the PDB files above. In addition, Yang et al. [21] and Knight et al. [22] reported that the X-ray crystal structures complexes with the inhibitors showed conformational changes upon binding of the inhibitors to the enzymes. In the IFD protocol, Glide docking with softened potential and side chain removal according to the B-factor was first carried out. Then, the results were clustered based on the scores and the descriptors. Next, the side chains in which residues are within a distance of 5 Å were predicted and the residues and the ligands in the complex pose were minimized by Prime. Subsequently, the protein and the ligand in the complex were re-docked.

## Results and discussion

### Pharmacophore model

**Fig. 1:**
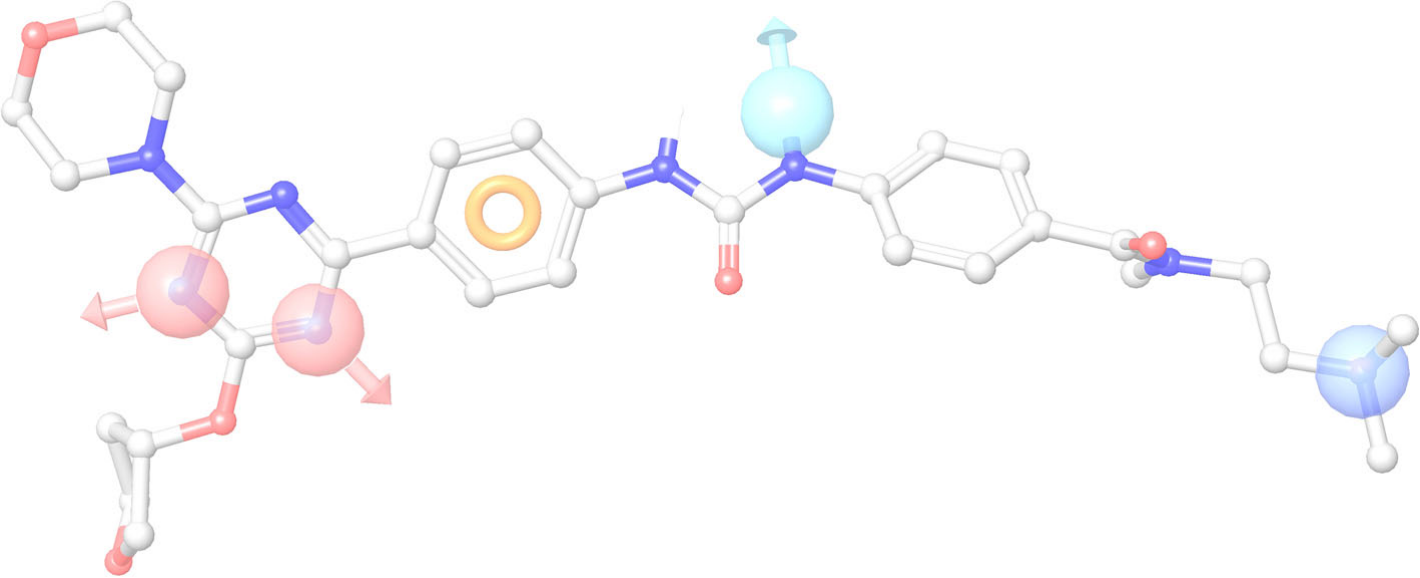
Pharmacophore model shown on MAC of PI3Kα. *Pink*: HB acceptor; *light blue*: HB donor; *purple*: positive feature; *orange ring*: aromatic ring

Pharmacophore models were the same for inhibitors for both PI3Kα and mTOR enzymes. In Fig. 1, MAC for PI3Kα was mapped with the pharmacophores of the highest scores. Pharmacophore models for PI3Kα and mTOR were AADPR, where A, D, P or R represents a hydrogen bond acceptor, a hydrogen bond donor, positive feature, or an aromatic ring, respectively. For both models, the two As were on the triazine nitrogens, and D was on the urea nitrogen. P was on the amine on the end of the molecules and R was on aromatic ring next to triazine.

### QSAR

Table S3 shows the observed and predicted activities. We randomly split the training and test set three times and calculated the average of the coefficients with uniform distribution of the activities of the compounds. The average and standard deviation of correlation coefficients of atom-based 3D-QSAR models for the training set (*R*^2^) and test set (*Q*^2^) for PI3Kα and mTOR were in *R*^2^ = 0.74 ± 0.05, *Q*^2^ = 0.65 ± 0.08 and *R*^2^ = 0.69 ± 0.04, *Q*^2^ = 0.56 ± 0.09, respectively. *Q*^2^ for both models were over 0.5.

The combined hydrophobic/non-polar and electron-withdrawing effects through the QSAR modeling for PI3Kα and mTOR are shown on the aligned structures for the five most active compounds in Figure S1 in Supplementary Material. MACs for both enzymes had combined hydrophobic/non-polar and electron-withdrawing effects at both ends of the compound structures including a morpholine and an amine moiety contributing to the inhibition activity.

### Binding modes

X-ray structures of PI3Kα and mTOR were reported only recently [21, 23]. The PI3Kγ- complex structure had been used previously to build homology models for the docking study of PI3Kα [6, 7]. We now conducted docking to study the ligand–protein interaction using the available PI3Kα and mTOR x-ray structures. Ligand interaction diagrams of the modeled structure complex are shown in Figure S2 and S3 in Supplementary Material for MACs and LACs with PI3Kα and mTOR respectively. The cut-off distance for interaction selection was set as 4 Å.

Based on the binding mode of MAC (CID 53379513) docked to PI3Kα (shown in Figure S2 (A) in Supplementary Material), the oxygen atom of the morpholine moiety formed a hydrogen bond (HB) with the backbone of Val 851. As shown in Figure S2 (A), the CH_2_ groups of the morpholine moiety are hydrophobic and surrounded by the hydrophobic residues, which agrees with the positive contributions of the combined hydrophobic/non-polar and electron-withdrawing effects on the morpholine group to the inhibition activity suggested by the QSAR modeling (shown in Figure S1). Besides, Figure S6 (B) shows that the morpholine and R1 group bind to non-polar region. Val 851 in PI3Kα corresponds to Val 882 in PI3Kγ which forms a HB with adenosine 5′-triphosphate (ATP) in the ATP-bound PI3Kγ crystal structure [24]. In addition, the urea moiety formed HBs with Lys 802 and Asp 810, the amide moiety formed a HB with His 936, and the amine moiety formed a HB with Asp 805. The hydrogen atom of this urea group was captured in the pharmacophore model as a hydrogen bond donor (Fig. 1). For Lys 802 in PI3Kα, its corresponding residue Lys 833 in PI3Kγ forms a HB with ATP [25]. Therefore, these HBs formed with Val 851 and Lys 802 are crucial for the ligand binding as an ATP-competitive drug. The other residues of PI3Kα, which correspond to the HB-forming residues in the ATP-bound PI3Kγ structure, are Ser 774, Glu 849, Gln 920, and Asp 933. Among them, Ser 774, Glu 849, and Asp 933 were observed within 4 Å from the ligand in the docked complex structure. Based on the previously reported binding mode of CID 53379513 docked to the conformation of PI3Kα derived through homology modeling, the morpholine moiety formed a HB with Val 851 and urea formed HBs with Lys 802 and Asp 810 [6]. When docking LAC (CID 46228569) to PI3Kα (shown in Figure S2 (B) in Supplementary Material), the binding mode was similar to that of the MAC. The difference was that Ser 774 was not observed within a 4 Å distance from the ligand comparing to the docked mode for MAC. In Figure S4 (A) in Supplementary Material, the binding mode of PKI-587 (CID 44516953) to PI3Kα showed all key HBs formed in the binding mode of MAC but Ser 774 was not observed within 4 Å from the ligand similarly to the observation in the docked mode for LAC. Molecular dynamics (MD) simulation was applied to docked structures (docked poses) to check the stability of the binding modes (Supplementary Material). The HBs formed between Val 851 and morpholine oxygen, between Lys 802 and urea oxygen, and between Asp 810 and urea hydrogens were observed with a high frequency during the 5 ns MD simulation, indicating the significance of their contributions for ligand-receptor binding and the stability of the docked modes. Estimated binding free energy with MM-GBSA for each binding mode was shown in Table S5 in Supplementary Material. For PI3Kα, the calculated binding free energy for PKI-587 was slightly higher than MAC, but the order of the estimated binding free energy correlated in general with the order of the experimental activities of the ligands.

**Fig. 2:**
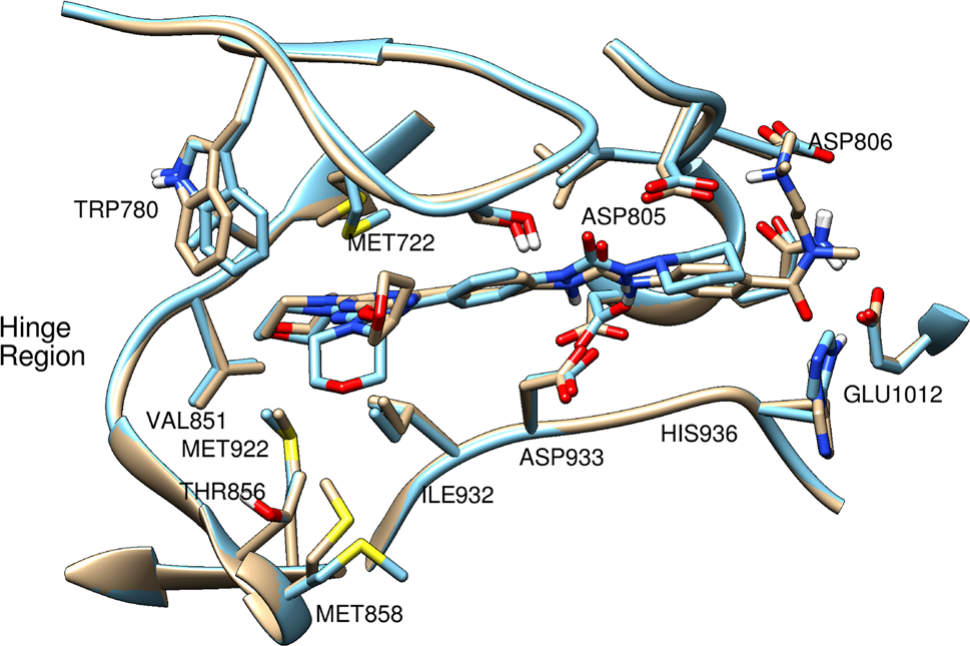
Overlay of the docked structure of MAC and LAC for PI3Kα. *Tan*: MAC and PI3Kα, *blue*: LAC and PI3Kα

Figure 2 shows the overlay of the binding sites in the docked modes for MAC and LAC to PI3Kα. Most parts of the MAC and LAC molecules were flat except those at the two ends. MAC is bigger in size and longer in shape comparing to LAC. The figure shows a region in MAC that was surrounded by the negatively charged or polar residues and bent upwards away from the flat region, which supported the suggestion from that pharmacophore model for positive feature at the end of this section of the molecule.

MAC made extra HBs with His 936 and Asp 805 fitting into the region consisting of the residue Asp 806, Gln 809, and Glu 1012. The position of the side chain of His 936 in the docked mode of PI3Kα bound with MAC slightly shifted towards MAC comparing to its position in the LAC bound mode. MAC also formed a HB with the amine at the end of the molecule to the opposite side of the morpholine group. The tetrahydrofuran group in MAC stuck out of the flat plane region (which consists of most fragments of the MAC) towards Met 722, and the sidechain of Trp 780 shifted slightly closer to the group than that in the LAC bound mode. In addition, the side chain of Met 858 flipped toward the tetrahydrofuran group in MAC.

Knight et al. [22] suggested that the selectivity of isoform-selective PI3K inhibitors resulted from the fit of the inhibitors into the pocket induced by the inhibitors between Met 804 and Trp 812 in PI3Kγ (corresponding residues, Met 772 and Trp 780 in PI3Kα). The quinazoline moiety of PIK-39 in the PI3Kγ crystal structure complex, occupies the pocket between Met 804 and Trp 812. It is perpendicular to the plane of the other part of the PIK-39 molecule. PIK-39 and other PI3K selective inhibitors tend to have the fragment sticking out of the plane structures while multi-target inhibitors tend to have solely flat structures. In terms of the molecular shape, both MAC and LAC for PI3Kα revealed mostly flat structures, which indicated that they were dual inhibitor candidates indeed.

For selective PI3Kα inhibitor design that uses the morpholino-triazine scaffold, substituting tetrahydrofuran to the polycyclic aromatic group in the MAC structure might induce Met 772 movement and create a pocket between Met 772 and Trp 780 like PIK-39. Knight et al. also reported that the potent selective inhibitors bound deeply into the ATP binding pocket by forming two HBs with the backbone of Val 882 and Glu 880 in PI3Kγ. Adding additional hydrogen bond donors on the morpholine moiety of MAC might build extra HB with the backbone of Glu 849 in PI3Kα and lead the compound to insert more deeply into the adenine pocket.

The binding mode of MAC (CID 44514208) docked to mTOR (Figure S3 (A) in Supplementary Material) showed HBs between morpholine and Val 2240, the urea moiety and Asp 2195, and the amine moiety and Asp 2360. In addition, the anime moiety formed a salt-bridge with Asp 2433. This agrees with the pharmacophore model where positive feature is at the end to the opposite side of the morphoine and triazine group (Fig. 1). Information of the corresponding residues in ATP-bound PI3Kγ for mTOR is provided in the Supplementary Material. The binding mode of LAC (CID 56683919) docked to mTOR showed a HB between urea and Lys 2187 but did not show any HB with Asp 2360 or salt-bridge with Asp 2433 (Figure S3 (B) in Supplementary Material). The binding mode for the PKI-587-mTOR model showed the same HBs formed as that in the MAC-mTOR model in addition to the HB formed between Lys 2187 and the oxygen of the urea. Additionally a salt-bridge formed between the amine moiety of PKI-587 and mTOR/Asp 2360 (Supplementary Material Figure S4 (B)). As observed in the model docked to PI3Kα, the morpholine moiety was in the hydrophobic pocket agreeing with the combined effects from QSAR modeling, and the urea hydrogen atom formed a HB, which was indicated in the pharmacophore model. Furthermore, the key HBs formed between Val 2240 and morpholine/oxygen, Lys 2187 and urea/oxygen, and Asp 2195 and urea/hydrogen atoms were observed with a high frequency during the 5 ns MD simulation (Supplementary Material). For mTOR, the estimated binding free energy of MAC and PKI-587 was both lower than that of LAC, though the estimated free energy of MAC was higher than that of PKI-587, suggesting more accurate free energy calculation may be needed.

**Fig. 3:**
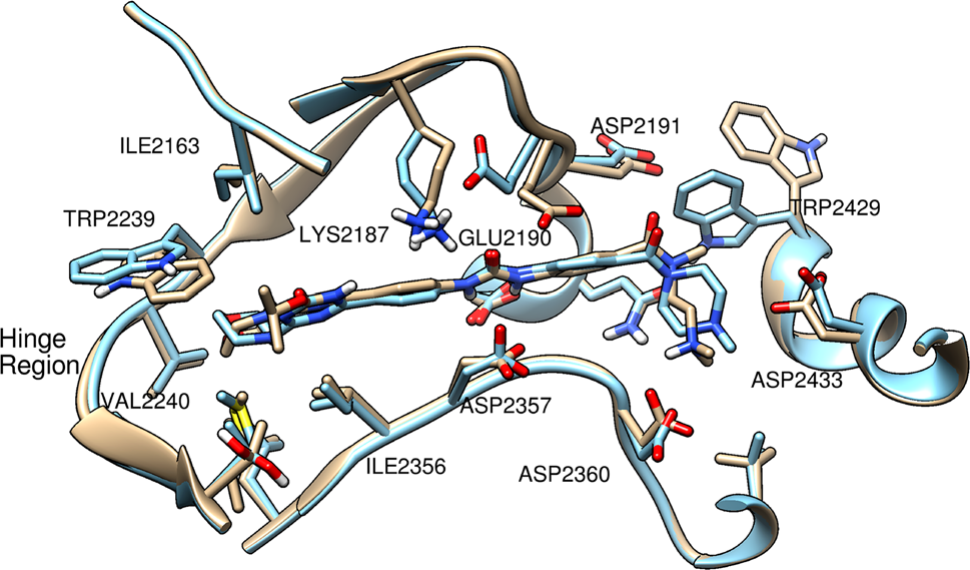
Overlay of the docked structure of MAC and LAC for mTOR. *Tan*: MAC and mTOR, *blue*: LAC and mTOR

Figure 3 shows the overlay of the binding sites in the models for MAC and LAC docked to mTOR. MAC and LAC occupied almost the same space in the docked model, and both structures were adopting mostly a flat plane conformation as observed in the docked modes of the MAC and LAC in PI3K. This flatness of the molecule is partially supported by the aromatic ring of pharmacophore in the pharmacophore model. However, a large difference was found between the complex structures with MAC and LAC in the position of Trp 2429. Trp 2429 in the LAC-mTOR complex was closer to the end of LAC while Trp 2429 in the MAC-mTOR complex opened up the space presumably to avoid the steric crash between the –N(CH_3_) group of MAC next to the carbonyl and Trp 2429. In addition, Glu 2190 of mTOR bound with MAC flipped to the opposite direction comparing to its orientation in the LAC-mTOR complex, and was close to the aromatic ring next to the urea group as if it attempts to close the space opened up by the shift of Trp 2429. The side chain of Trp 2239 in the MAC-mTOR complex shifted slightly towards the –OC(CH_3_)_2_ group of triazine. For both MAC and LAC, the end part of the molecule which is on the other side of morpholine bent towards the region consisting of Asp 2360 and Asp2433 and stuck out of the flat plane of the ligand molecule (in Fig. 3 going downwards).

Yang et al. [21] reported that a mTOR selective inhibitor, Torin2, had stacking effect between its aromatic ring and Trp 2239, and that Torin2 is surrounded by a pocket consisting of Ile 2163, Pro 2169, and Leu 2185. Neither MAC nor LAC in the docked structure showed a similar stacking effect from the aromatic ring with Trp 2239. However, the docking model suggested that such stacking effect may be formed if MAC and LAC would insert deeper into the hinge region, or if Trp 2239 would shift to certain extent towards the ligands. This analysis suggests that introducing a polycyclic aromatic ring group at the position of the extended flat plane of the ligand’s structure may be critical for improving inhibition selectivity through stacking effects. Yang et al. also reported that another mTOR selective inhibitor, PP242, showed conformational change of the hydrophobic pocket upon its binding to mTOR, which involved residue Tyr 2225, Gln 2223, and Leu 2354. Among those residues, they suggested Leu 2354 was the key residue for the compound’s selectivity towards mTOR over PI3Ks because the PI3Ks and mTOR differ in sequence at that position of Leu 2354 in mTOR where phenylalanine is found in PI3Ks. However, the docked models for MAC-mTOR and LAC-mTOR did not show significant conformational changes as those caused by the binding of the selective inhibitor PP242 to mTOR, which may help to explain why compounds including MAC and LAC were showing dual inhibition activity rather than selectively favoring mTOR or PI3K.

Comparing the docked modes for PKI-587 and MAC to PI3Kα and mTOR, PKI-587 binds more similarly to PI3Kα and mTOR, while larger differences were observed in the models for MAC and the enzymes. In MAC- PI3Kα, MAC formed HBs with Lys 802 and His 936 but no salt-bridge was observed. Conversely, in MAC-mTOR, no HB was formed between MAC and the corresponding residues of mTOR though a salt-bridge was formed. When looking into the docked modes of PKI-587 to PI3Kα and mTOR, while a salt-bridge was found in PKI-587-mTOR but not in PKI-587-PI3Kα, two binding modes otherwise showed a similar HB pattern. This “reduced” difference may help to explain the dual inhibition activity of PKI-587 and shed a light for designing dual vs. selective inhibitors.

Both MAC and LAC docked to PI3Kα by occupying a larger space in the pocket compared to the binding modes of mTOR inhibitors. In the docked structures for MAC-PI3Kα and LAC-PI3Kα, the areas of the receptor surface within 4 Å from the ligand were 1661 and 1344 Å^2^ respectively while for MAC-mTOR and LAC-mTOR the areas were 1256 and 961 Å^2^. The overlay of the binding sites in the modes for MAC and LAC docked to PI3Kα and mTOR are shown in Figs. 2 and 3 respectively. The bound structures of MAC and LAC aligned well with each other. The areas of the receptor surface within 4 Å from PKI-587 in the modes docked to PI3Kα and mTOR were 1461 and 1100 Å^2^, respectively, which fall in between the surface area obtained from the respective MACs-receptor and LACs-receptor docked structures. It is noteworthy that the order of receptor surface areas in the ligand-receptor docked structures correlated to certain extent with the order of ligand inhibition activities. Figure S6 (A) in Supplementary Material shows the superimposed binding site surface of PI3Kα and mTOR bound with PKI-587, which indicated that PI3Kα possessed a larger opening space in the binding pocket where morpholine and the R1 group of the ligand bind, which opens up toward its C-terminal section.

Overall the docked modes of the inhibitors for both PI3Kα and mTOR indicated that inhibitors with the morpholino-triazine scaffold had mostly the same inhibition mechanism. MACs mainly had flat structures except the parts at the terminal of the compounds. They formed HBs between the morpholine moiety and a valine in the receptor’s hinge region, the urea moiety and an aspartic acid of the receptor, and the amine moiety at the terminal part of the ligand and the aspartic acid in the region of the receptor that consists of negatively charged or polar residues. LACs docked to the enzymes by forming HBs only at the morpholine and urea moieties. This relative shortage of HBs in the LAC complexes seems to be the primary reason accounting for the bioactivity difference. A larger difference between MAC-PI3Kα and MAC-mTOR lied mainly in the shape of the binding sites. PI3Kα possessed a larger opening of binding site. In addition, the docked conformation of MAC in PI3Kα bent towards the N-terminal section (upwards in Fig. 2) while that in mTOR bent towards the C-terminal section (downwards in Fig. 3). This difference might be utilized as another strategy for the design of selective inhibitors, which is in addition to the suggestion for introducing a polycyclic aromatic group to gain ring stacking effects with Trp 780 for PI3Kα and Trp 2239 for mTOR depending on the direction of each tryptophan in the respective enzyme target. The receptor surface in the area within 4 Å from the ligand was larger in models for PI3Kα than that in models for mTOR. Similar binding modes of PKI-587 to PI3Kα and mTOR were observed, which may be one of the key factors to the success of its dual inhibition bioactivity against both receptors.

## Conclusion

To understand the molecular basis of a dual inhibitor of PI3K/mTOR that contains the morpholino-triazine moiety, we constructed pharmacophore and QSAR models using experimentally identified PI3K and mTOR inhibitors with the scaffold of morpholino-triazine. We also studied the binding modes of MACs, LACs, and PKI-587, by docking them to PI3Kα and mTOR. The pharmacophore models were the same for PI3Kα and mTOR. It appeared that MACs for PI3Kα and mTOR shared the same mechanism of the inhibition as suggested by the QSAR model and docking study. Binding modes from the docking study revealed that both MACs and LACs mostly adopted a flat plane conformation except the parts at the end of the molecules. Based on the docked structures to both PI3Kα and mTOR, the binding modes of MAC and LAC differed by one HB formed with amine on the one end of the ligand molecule. Similar binding modes were observed for PKI-587 docked to PI3Kα and mTOR, which might be a key principle for achieving dual inhibition bioactivity. The analysis also suggested the importance of the space surrounding the ligand in the binding pocket and of the slight difference of the shape of the binding site between PI3Kα and mTOR for the design of selective inhibitors.

## Electronic supplementary material

Below is the link to the electronic supplementary material.

Supplementary material 1 (PDF 4201 kb)

## Abbreviations

HB: Hydrogen bond
mTOR: Mammalian target of rapamycin
PI3K: Phosphatidylinositol-4,5-bisphosphate 3-kinase
QSAR: Quantitative structure–activity relationship
MAC: Most active compound
LAC: Least active compound
