# Supplementary material for: Structural insights of a PI3K/mTOR dual inhibitor with the morpholino-triazine scaffold
Source: J Comput Aided Mol Des. 2016 Mar 8;30:323–30. doi: 10.1007/s10822-016-9905-4 (PMC4833818; doi:10.1007/s10822-016-9905-4)
Supplement: Supplementary file 1 — Supplementary material 1 (PDF 4201 kb) [file 10822_2016_9905_MOESM1_ESM.pdf]

Supplementary Material

TITLE

Structural Insights of a PI3K/mTOR Dual Inhibitor with the morpholino-triazine scaffold

JOURNAL NAME

Journal of Computer-Aided Molecular Design

AUTHOR NAMES

Takako Takeda, Yanli Wang, Stephen. H. Bryant.*

AUTHOR AFFILIATION

National Center for Biotechnology Information, National Library of Medicine, National Institutes of Health, Bethesda, MD 20894

CORRESPONDING AUTHOR:

Yanli Wang

Email: ywang@ncbi.nlm.nih.gov, Tel/fax: 1-301-435 -7811/1-301-435-7794

Table S1-1 SAR-table (core structure)

1	2
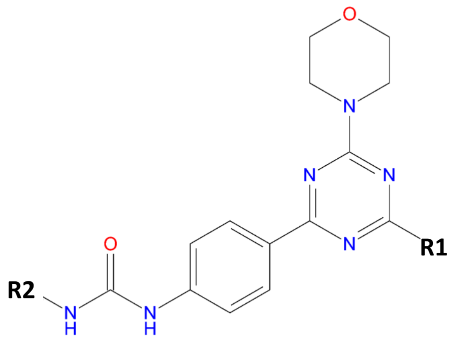	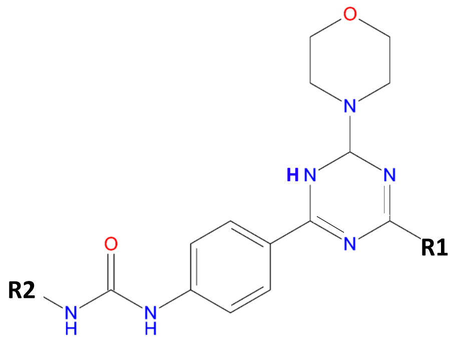

Table S1-2 SAR-table (continued)

Structure Name	R1	R2	Core Number	pIC50 PI3K	pIC50 mTOR
44473371	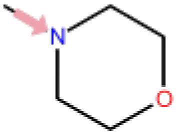	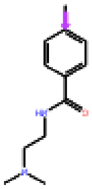	1	9.40	9.30
45379226	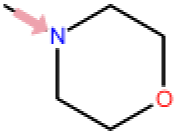	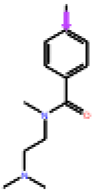	1	9.40	9.22
44516953	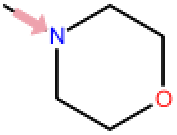	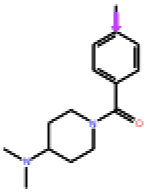	1	9.40	8.80
45379224	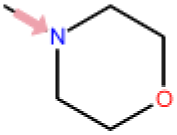	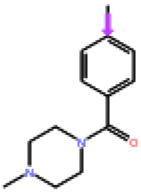	1	9.30	8.85
44516636	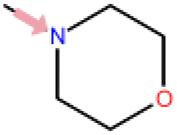	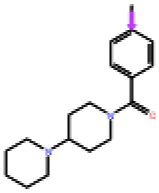	1	9.22	9.15
44516635	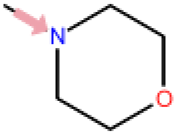	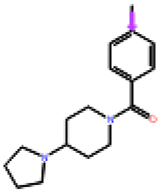	1	9.15	9.10
44515084	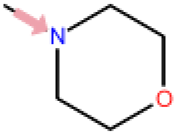	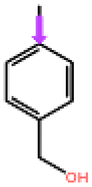	1	9.00	8.72
44516310	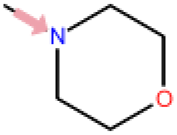	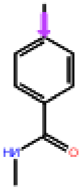	1	8.82	9.15
44515083	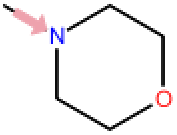	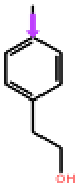	1	8.82	8.74

Table S1-3 SAR-table (continued)

Structure Name	R1	R2	Core Number	pIC50 PI3K	pIC50 mTOR
44513885	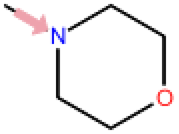	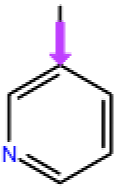	1	8.77	8.92
44513887	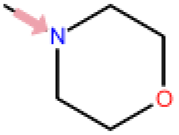	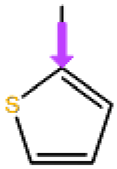	1	8.70	8.77
44513884	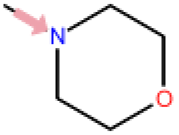	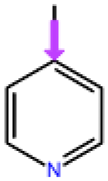	1	8.52	8.92
44513886	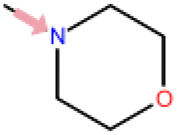	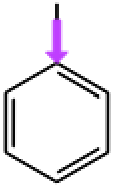	1	8.52	8.41
44516307	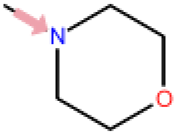	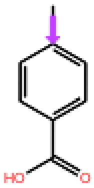	1	8.22	8.82
44514201	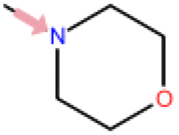	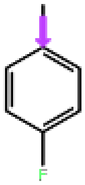	1	8.19	8.41
45379223	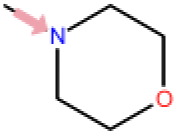	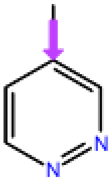	1	7.96	8.64
44515696	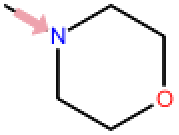	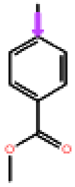	1	7.90	8.60
44513888	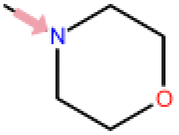	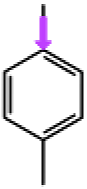	1	7.78	8.20

Table S1-4 SAR-table (continued)

Structure Name	R1	R2	Core Number	pIC50 PI3K	pIC50 mTOR
44514203	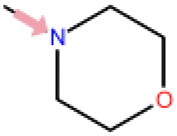	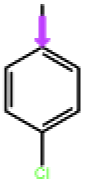	1	7.71	8.17
44514205	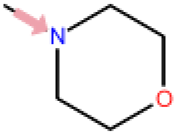	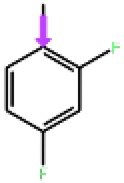	1	7.22	8.03
44514481	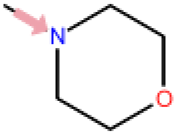	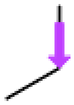	1	6.61	8.00
45379222	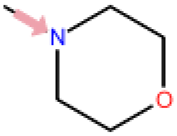	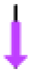	1	6.46	7.88
46228569	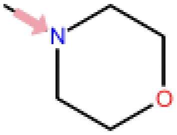	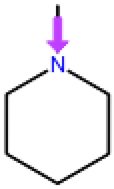	1	5.82	6.34

Table S1-5 SAR-table (continued)

Structure Name	R1	R2	Core Number	pIC50 PI3K	pIC50 mTOR
53379513	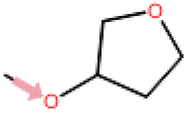	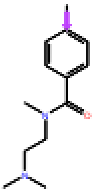	1	9.70	9.15
56683917	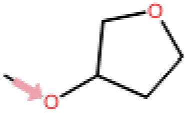	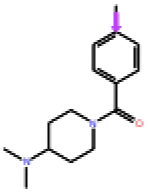	1	9.00	8.96
56660109	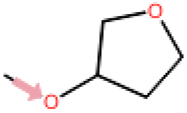	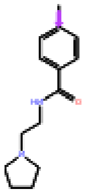	1	8.85	9.52
56673952	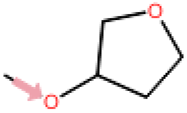	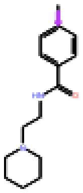	1	8.85	9.40
56683918	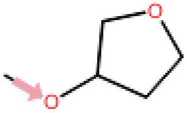	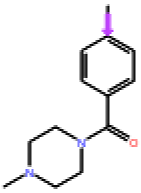	1	8.77	8.77
56660108	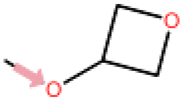	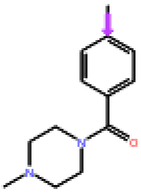	1	9.22	8.52
44514208	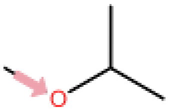	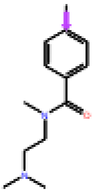	1	9.00	9.52
44514210	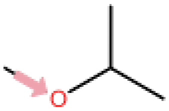	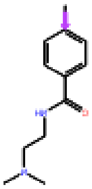	1	8.52	9.40
44517288	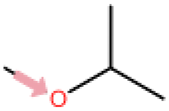	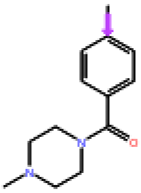	1	8.52	8.89

Table S1-6 SAR-table (continued)

Structure Name	R1	R2	Core Number	pIC50 PI3K	pIC50 mTOR
56667023	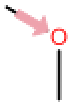	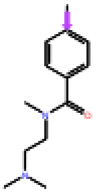	1	9.40	8.11
56677282	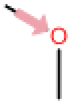	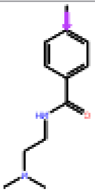	1	9.22	8.25
56683916	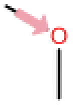	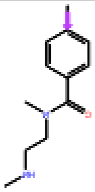	1	9.10	7.84
56677283	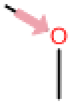	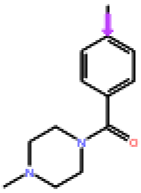	1	8.92	7.65
56663573	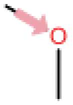	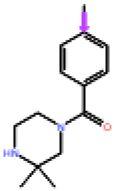	1	8.85	7.73
56670464	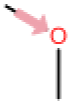	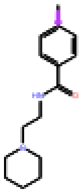	1	8.70	8.09
44548482	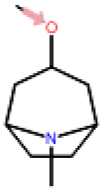	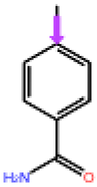	1	8.49	8.28
56683919	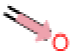	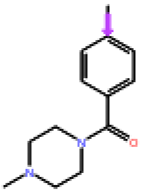	2	7.82	5.92

Table S2 Examples of activity cliffs

(A)

Structure Name	R1	R2	Core Number	pIC50 PI3K	pIC50 mTOR
44515696	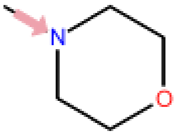	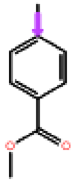	1	7.90	8.60
44516953	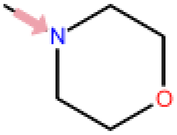	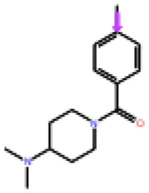	1	9.40	8.80

(B)

Structure Name	R1	R2	Core Number	pIC50 PI3K	pIC50 mTOR
56667023	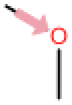	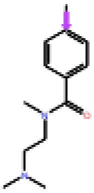	1	9.40	8.11
44514208	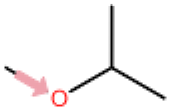	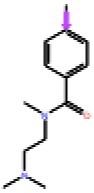	1	9.00	9.52

(C)

Structure Name	R1	R2	Core Number	pIC50 PI3K	pIC50 mTOR
46228569	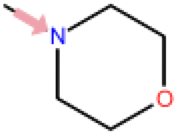	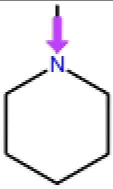	1	5.82	6.34
44513886	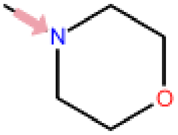	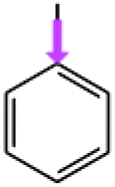	1	8.52	8.41

Table S3 Observed and predicted activities

	PI3K		mTOR	
CID	observed	predicted	observed	predicted
44473371	9.40[a]	9.66	9.30[a]	9.30
44513884	8.52	8.14	8.92	8.74
44513885	8.77	8.28	8.92	8.17
44513886	8.52	8.18	8.41	8.22
44513887	8.70	8.26	8.77	8.55
44513888	7.78	8.29	8.20	8.72
44514201	8.19	8.25	8.41	8.65
44514203	7.71	8.30	8.17	8.71
44514205	7.22	7.31	8.03	8.13
44514208	9.00	9.05	9.52[a]	9.36
44514210	8.52	9.10	9.40[a]	9.42
44514481	6.61	7.69	8.00	8.04
44515083	8.82	7.78	8.75	8.24
44515084	9.00	7.69	8.72	8.80
44515696	7.90	7.50	8.60	8.36
44516307	8.22	8.36	8.82	8.51
44516310	8.82	8.57	9.16	8.36
44516635	9.16	9.20	9.10	9.14
44516636	9.22	9.35	9.16	8.96
44516953	9.40[a]	9.27	8.80	8.68
44517288	8.52	8.50	8.89	9.01
44548482	8.50	8.40	8.28	8.95
45379222	6.46[b]	7.02	7.88	7.91
45379223	7.96	8.16	8.64	8.70
45379224	9.30[a]	8.66	8.85	8.93
45379226	9.40[a]	9.41	9.22	8.67
46228569	5.82[b]	6.35	6.34[b]	6.41
53379513	9.70[a]	8.95	9.16	9.33
56660108	9.22	8.95	8.52	8.43
56660109	8.85	9.47	9.52[a]	9.54
56663573	8.85	8.78	7.73	7.73
56667023	9.40[a]	9.60	8.11	7.99
56670464	8.70	8.96	8.09	8.17
56673952	8.85	9.01	9.40[a]	8.55
56677282	9.22	9.39	8.25	8.34
56677283	8.92	8.50	7.65	8.54
56683916	9.10	9.02	7.84	8.17
56683917	9.00	8.93	8.96	8.06
56683918	8.77	—-	8.77	—-
56683919	7.82	—-	5.92[a]	—-

a: most active compound
b: least active compound

Table S4 QSAR coefficient

		average	sd
PI3Kα	Q^2	0.65	0.08
	R^2	0.74	0.05

		average	sd
mTOR	Q^2	0.56	0.09
	R^2	0.69	0.04

Table S5 MM-GBSA results

		pIC50	dG
PI3Kα	LAC (46228569)	5.82	-113.61
	PKI-587	9.40	-125.17
	MAC (53379513)	9.70	-123.75

		pIC50	dG
mTOR	LAC (56683919)	7.84	-122.30
	PKI-587	9.38	-151.39
	MAC (44514208)	9.52	-136.76

Table S6 Molecular weight of the compounds (g/mol)

CID	MW
44473371	575.66
44513884	462.50
44513885	462.50
44513886	461.52
44513887	467.54
44513888	475.54
44514201	479.51
44514203	495.96
44514205	497.50
44514208	562.66
44514210	548.64
44514481	413.47
44515083	505.57
44515084	491.54
44515696	519.55
44516307	505.53
44516310	518.57
44516635	641.76
44516636	655.79
44516953	615.73
44517288	560.65
44548482	558.63
45379222	399.45
45379223	463.49
45379224	624.13
45379226	626.15
46228569	468.55
53379513	590.67
56660108	574.63
56660109	602.68
56663573	546.62
56667023	534.61
56670464	560.65
56673952	616.71
56677282	520.58
56677283	532.59
56683916	520.58
56683917	616.71

(A)

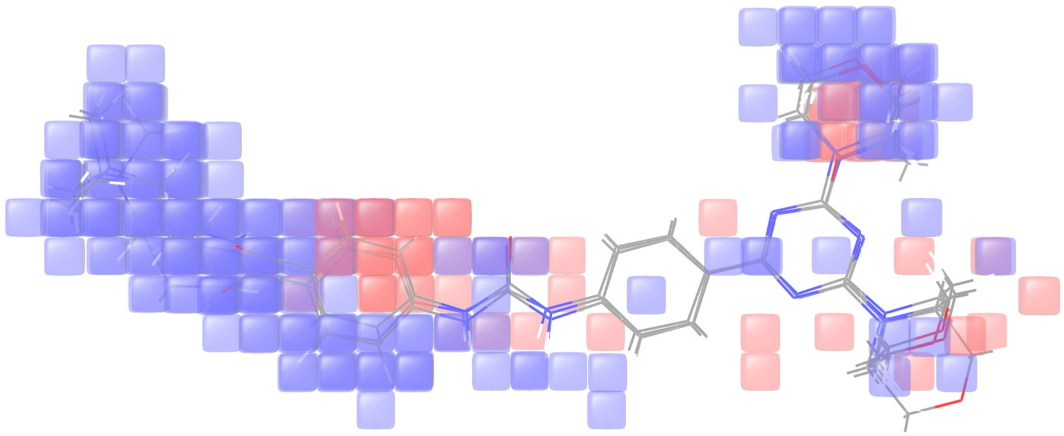

(B)

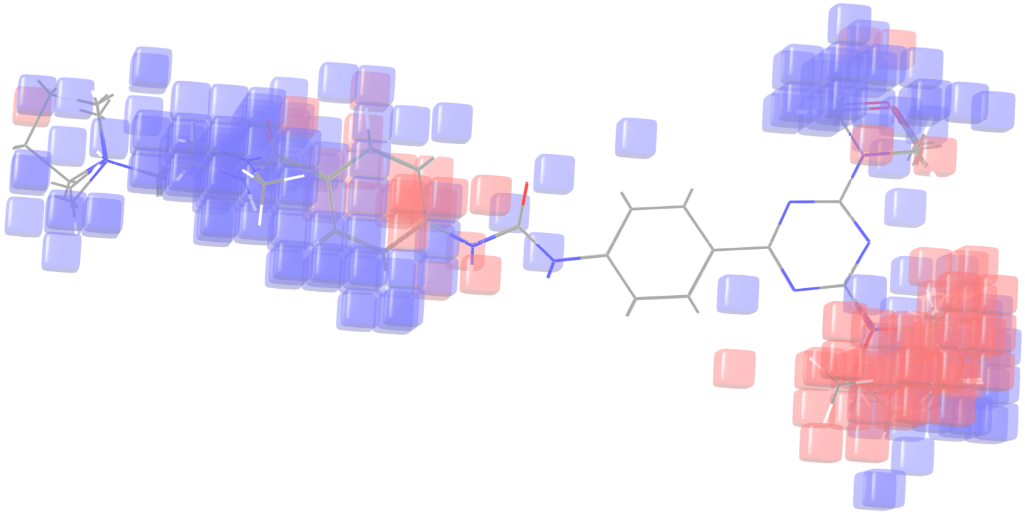

Figure S1 Combined effects and the 2D structures on aligned actives

(A) for PI3Kα (B) for mTOR

Blue cubes: positive contribution to the inhibitive effects, red cubes: negative contributions.

.

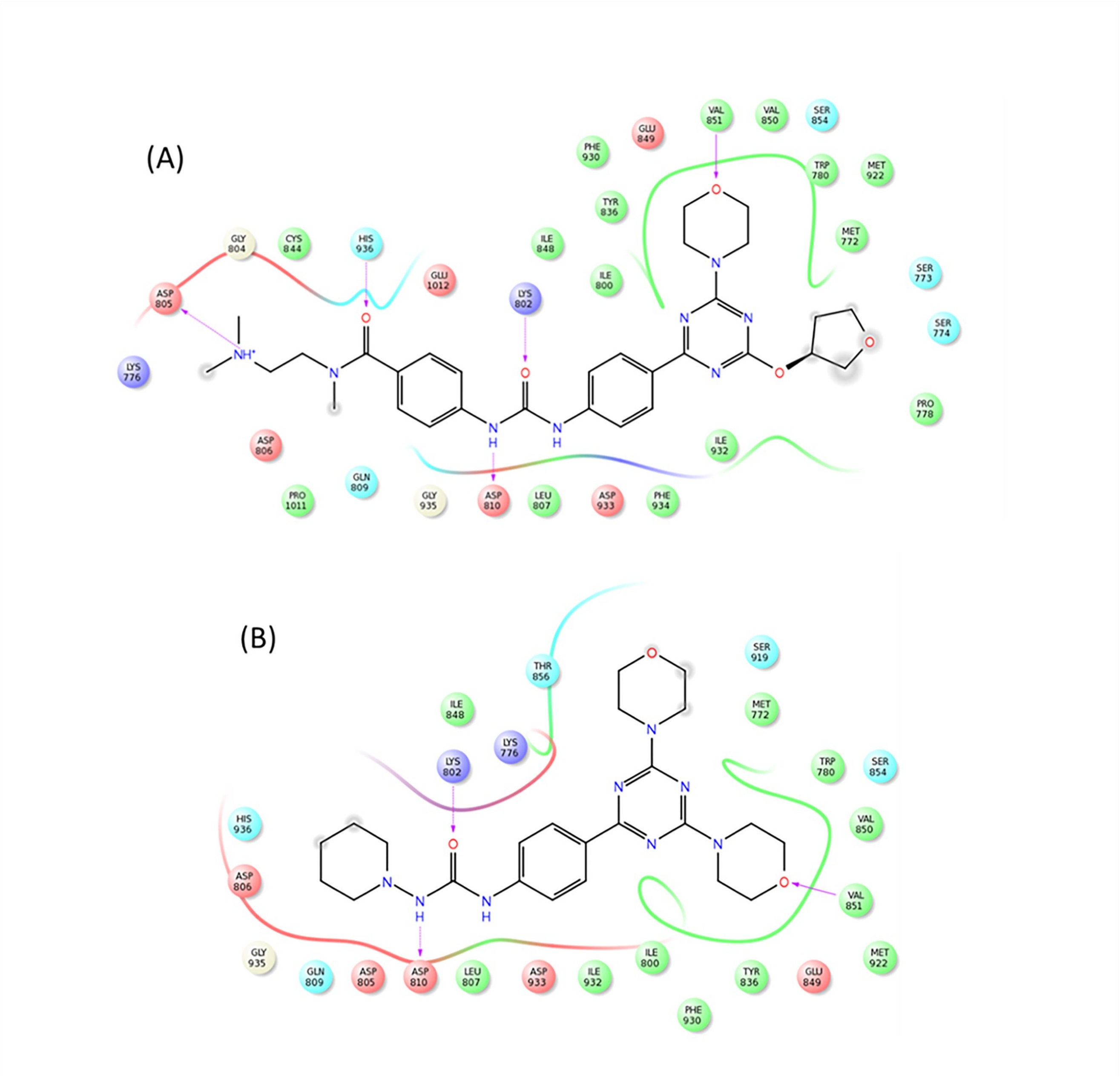

Figure S2 Ligand interaction diagram showing residues of PI3Kα near ligands in docked complex structure for the MAC and LAC

The cut-off distance was 4 Å. (A) CID 53379513 (B) CID 46228569. Red circle: charged residue (negative), purple circle: charged (positive), blue: polar, green: hydrophobic, gray cloud: solvent exposure, purple arrow: hydrogen bond. Colored curved lines around ligands are protein "pocket" and the color represents the color of the closest residue.

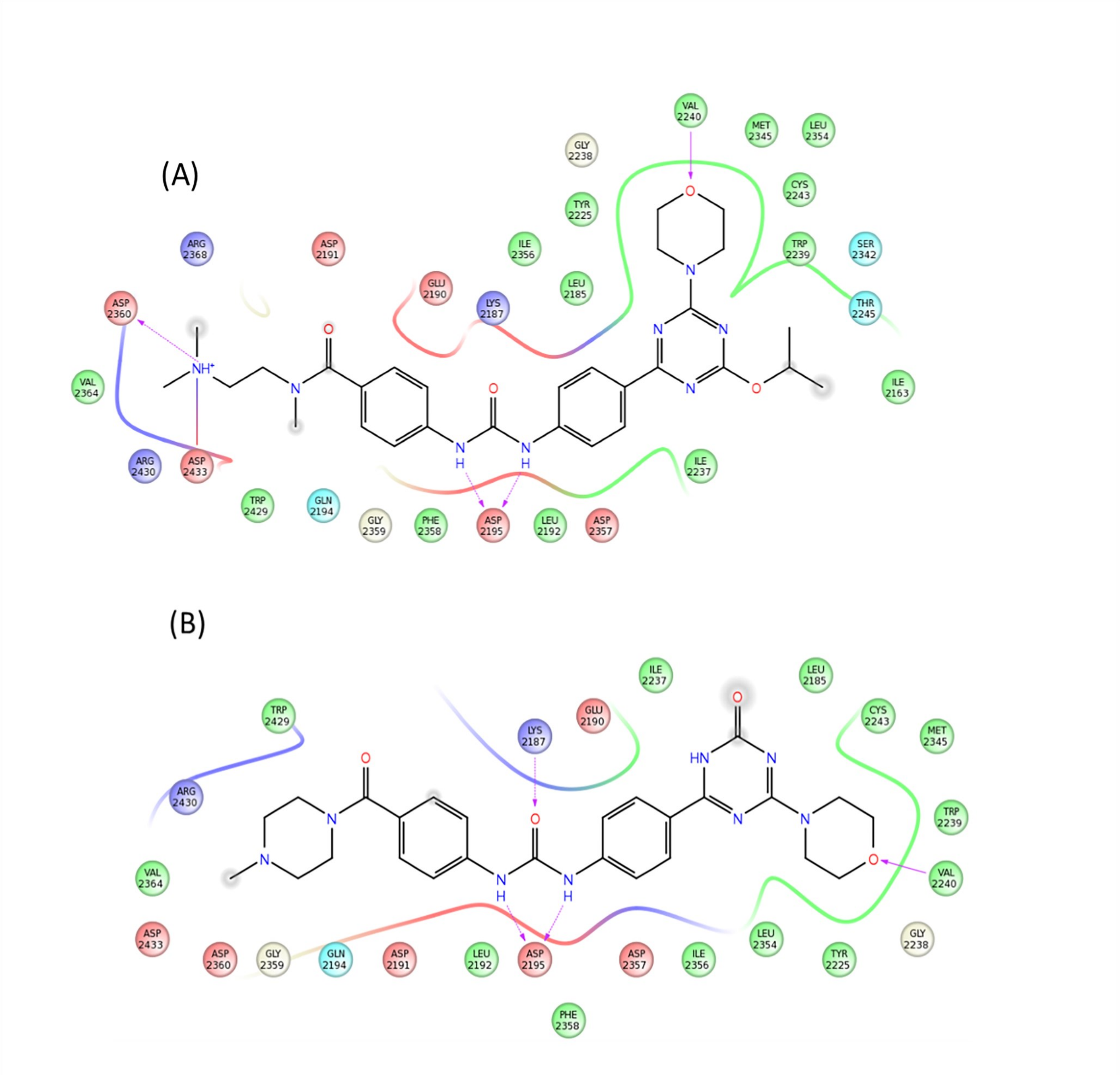

Figure S3 Ligand interaction diagram showing residues of mTOR near ligands in docked complex structure for the MAC and the LAC

(A) CID 44514208 (B) CID 56683919. The cut-off distance was 4 Å. Red circle: charged residue (negative), purple circle: charged (positive), blue: polar, green: hydrophobic, gray cloud: solvent exposure, purple arrow: hydrogen bond. Purple line: salt bridge. Colored curved lines around ligands are protein “pocket” and the color represents the color of the closest residue.

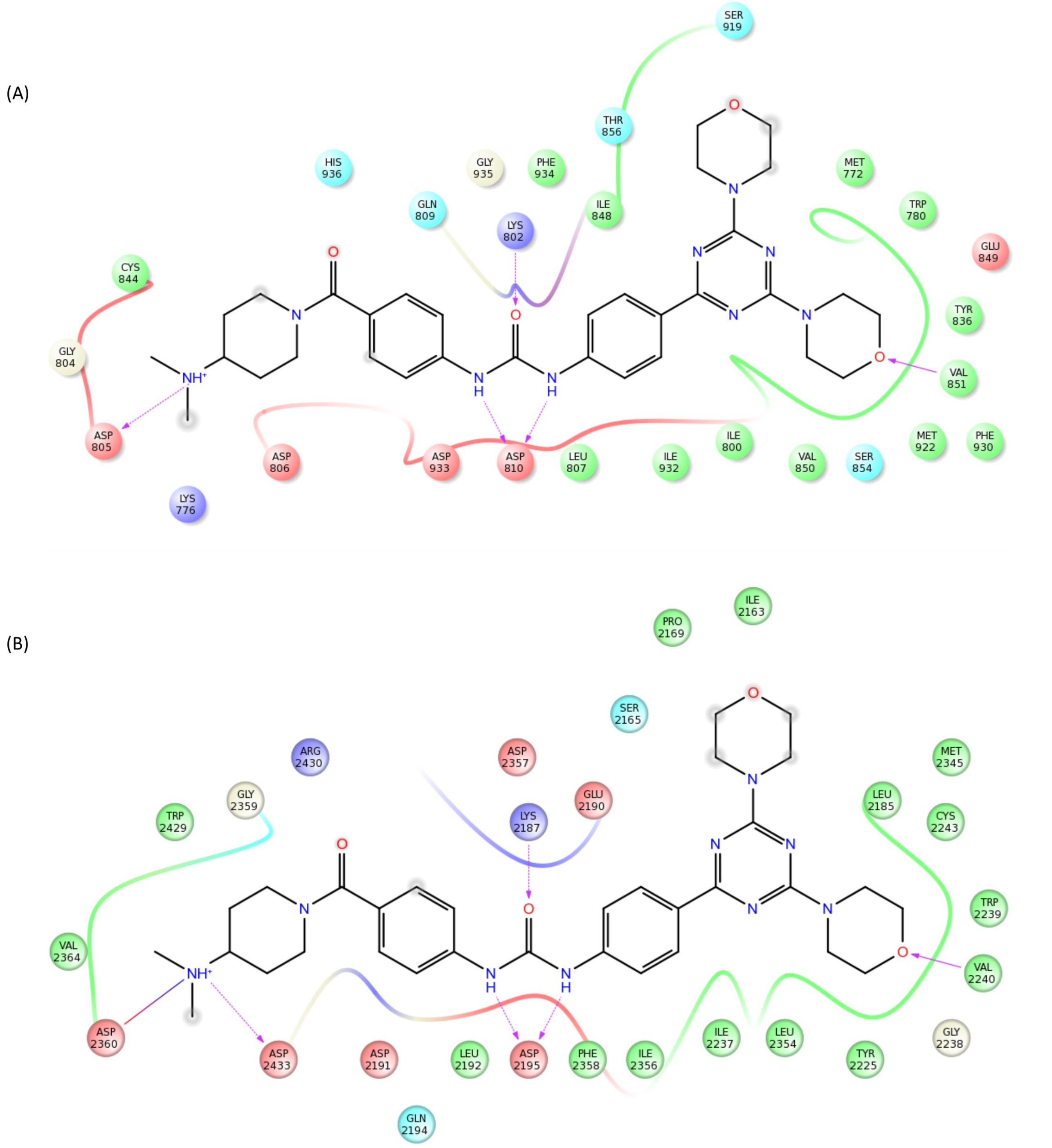

Figure S4 Ligand interaction diagram showing residues near PKI-587 in docked complex structure

(A) for PI3Kα (B) for mTOR
The cut-off distance was 4 Å. Red circle: charged residue (negative), purple circle: charged (positive), blue: polar, green: hydrophobic, gray cloud: solvent exposure, purple arrow: hydrogen bond, purple line: salt bridge. Colored curved lines around ligands are protein "pocket" and the color represents the color of the closest residue.

A)

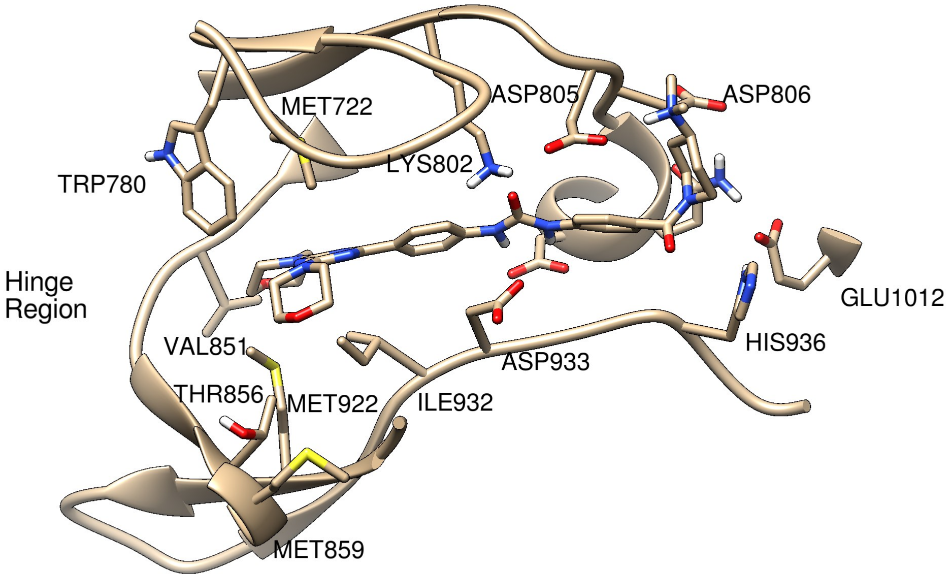

B)

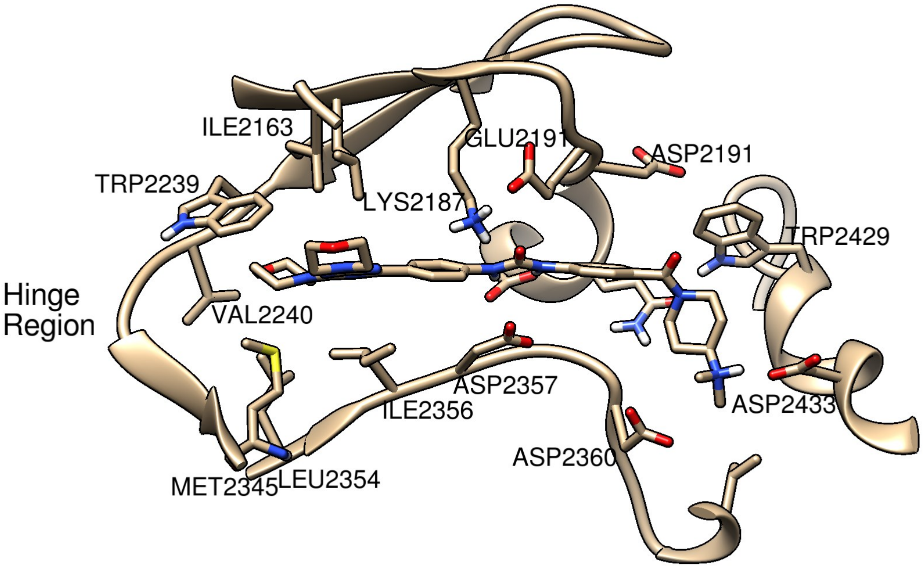

Figure S5 Docked structures of PKI-587

(A) for PI3Kα (B) for mTOR

(A)

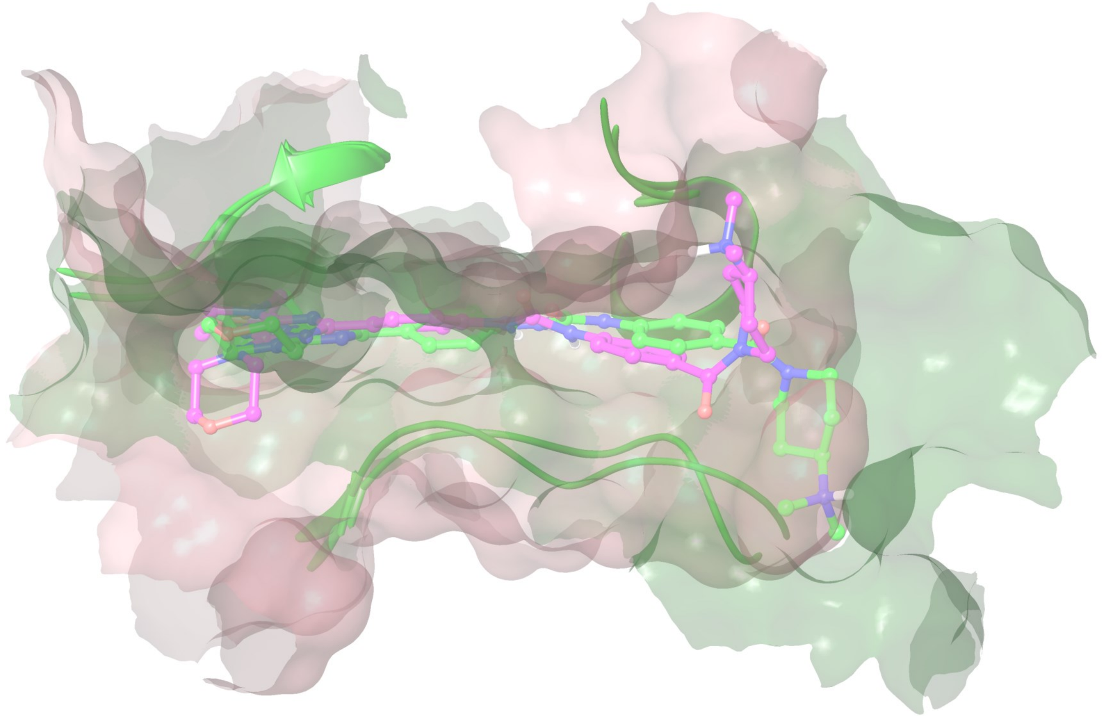

(B)

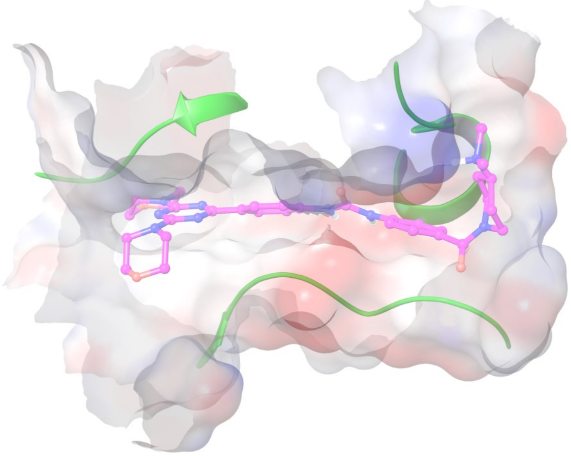

(C)

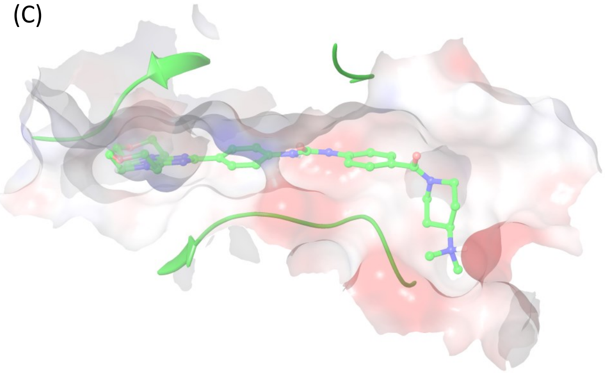

Figure S6 Binding site surface

(A) Image of superimposed binding site surface of the docked structures of PKI-587 for PI3Kα and mTOR Pink : PKI-587 with PI3Kα green : : PKI-587 with mTOR (B) Electrostatic representation of binding site surface of the docked structures of PKI-587 for PI3Kα blue:positively charged surface, red: negatively charged, white: non-polar. (C) Electrostatic representation of binding site surface of the docked structures of PKI-587 for mTOR Blue:positively charged ,red: negatively.

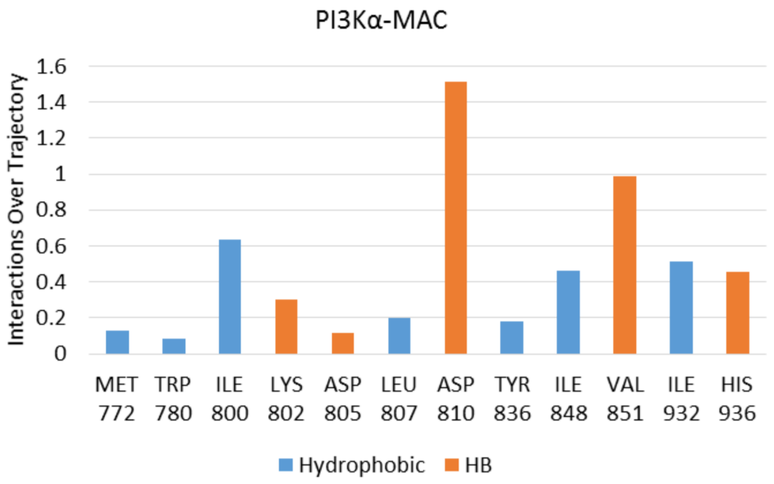

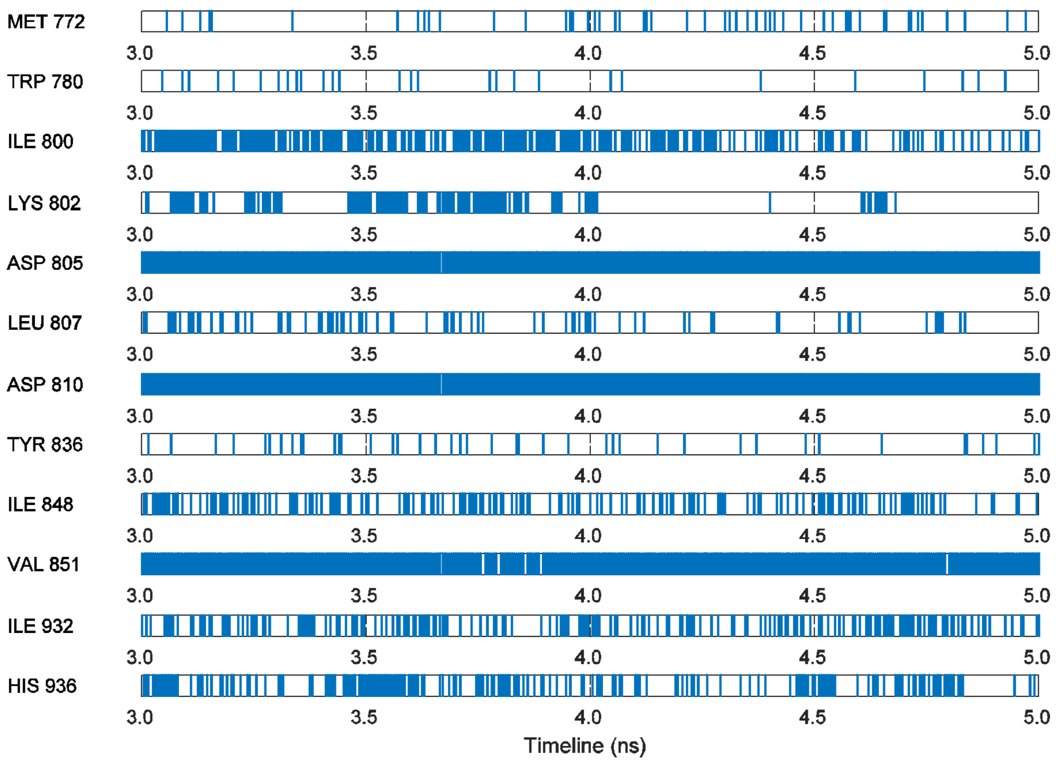

Figure S7 PI3Kα-MAC Interaction over the course of trajectory and timeline representation in MD (3ns-5ns)

(A) PI3Kα-MAC Interaction over the course of trajectory (Hydrophobic and HB) in MD during 3ns to 5ns. A value 0.6 indicates that for 60% of the simulation time the interaction existed. Values more than 1.0 indicates that the residue form multiple contacts (B) PI3Kα-MAC timeline in MD during 3ns to 5ns**.**

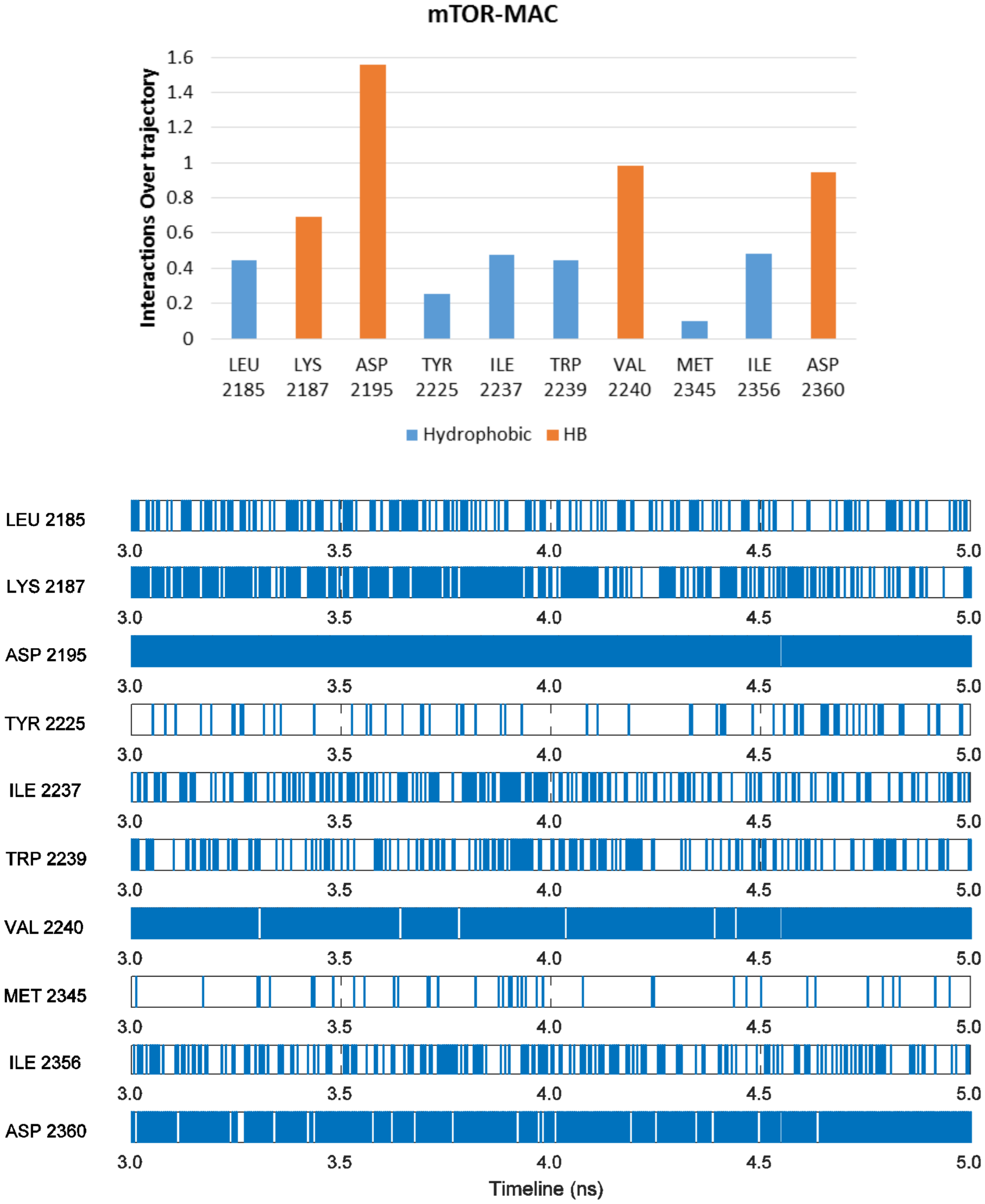

Figure S8 mTOR-MAC Interaction over the course of trajectory and timeline representation in MD (3ns-5ns)

(A) mTOR-MAC Interaction over the course of trajectory (Hydrophobic and HB) in MD during 3ns to 5ns. A value 0.6 indicates that for 60% of the simulation time the interaction existed. Values more than 1.0 indicates that the residue form multiple contacts. (B) mTOR-MAC timeline in MD during 3ns to 5ns.

Molecular Dynamics Simulation

Molecular dynamics (MD) simulations were applied to docked structures (docked poses) using Desmond (Schrödinger, LLC, New York, NY, 2013) to check the stability of the binding modes. The system was built by the system builder module in Maestro with the TIP3P water model and OPLS2005 force field. The initial coordinates for the MD computations were taken from the docked results. Na+ counter ions were added to neutralize the system and then 0.15M of NaCl was added. The total numbers of atoms in the system were 127426, 127464, 127459 for structure complexes, CID56683919 and mTOR, CID44514208 and mTOR, and PKI-587 and mTOR, respectively; and the total number of atoms were 119384, 118361, and 120152 for PKI-587 and PI3Kα, CID 46228569 and PI3Kα, and CID 53379513 and PI3Kα, respectively. The shape of the simulation box was orthorhombic. The box size was calculated using a buffer distance of 10 Å between the solute structures and the simulation box boundary, and the volume of the box was minimized subsequently. The systems were minimized and pre-equilibrated by the Desmond default relaxation protocol which consists of a series of minimizations and short molecular dynamics simulations. The system was equilibrated and then moved to the production stage following the relaxation process. The Nose-Hoover chain thermostat method was used with relaxation time of 1ps to maintain the temperature at 300K and the Martyna-Tobias-Kline barostat method was used with relaxation time of 2 ps. The short-range coulombic interactions were truncated with cut-off value of 9 Å. The long-range coulombic interactions were controlled by the Smooth particle mesh Ewald method with tolerance of $1e^{-9}$. Total simulation times were 5 ns for all simulations. The trajectory recording interval was 10 ps.

Sequence alignment (BLASTP 2.3.0+ RESULTS [1])

BLASTP 2.3.0+with default parameters, Uniprot amino acid sequence of the catalytic domain of PI3Ka (sp|P42336|797-1068) and mTOR (sp|P42345|2182-2516) were aligned.

Query: sp|P42345|2182-2516 (mTOR)

Subject: sp|P42336|797-1068 ((PI3Kα)

Alignment statistics for match #1

Score Expect Method **Identities** Positives Gaps

58.2 bits(139) 3e-14 Compositional matrix adjust. **53/201(26%)** 89/201(44%) 46/20122%)

```
Query  4    LLKGHEDLRQDERVMQLFGLVNTLLANDPTSLRKNLSIQRYAVIPLSTNSGLIGWVPHCD  63

            + K  +DLRQD   +Q+  ++  +  N    LR    +  Y  + +    GLI  V +

Sbjct  4    IFKNGDDLRQDMLTLQIIRIMENIWQNQGLDLR----MLPYGCLSIGDCVGLIEVVRNSH  59


Query  64   TLHALIRDYREKKKILLNIEHRIMLRMAPDYDHLTLMQKVEVFEHAVNNTAGDDLAKLLW  123

            T+              + I+ +  L+ A  ++  TL Q                     W

Sbjct  60   TI--------------MQIQCKGGLKGALQFNSHTLHQ---------------------W  84


Query  124  LKSPSSEVWFDRRTN-YTRSLAVMSMVGYILGLGDRHPSNLMLDRLSGKILHIDFGDCFE  182

            LK  +    +D   + +TRS A   +  +ILG+GDRH SN+M+ +  G++ HIDFG   +

Sbjct  85   LKDKNKGEIYDAAIDLFTRSCAGYCVATFILGIGDRHNSNIMV-KDDGQLFHIDFGHFLD  143


Query  183  VAMTREKFP---EKIPFRLTR  200

                ++KF    E++PF LT+

Sbjct  144  --HKKKKFGYKRERVPFVLTQ  162
```

Range 2: 261 to 271GraphicsNext MatchPrevious MatchFirst Match

Alignment statistics for match #2

Score Expect Method Identities Positives Gaps

15.4 bits(28) 1.9 Compositional matrix adjust. 5/11(45%) 6/11(54%) 0/11(0%)

```
Query  58   WVPHCDTLHAL  68

            W+ H    HAL

Sbjct  261  WIFHTIKQHAL  271
```

1)Altschul SF, Madden TL, Schäffer AA, et al. (1997) Gapped BLAST and PSI-BLAST: a new generation of protein database search programs. Nucleic Acids Res 25:3389–3402. doi: 10.1093/nar/25.17.3389

Information of corresponding residues in ATP-bound PI3Kɣ for mTOR

Val 2240 is the corresponding reside of Val 882 in PI3Kɣ. Asp 2195 is observed as forming HB with PI-103, The other dual inhibitor , in the crystal structure of mTOR–PI-103 complex [2]. The other residues corresponding to HB forming residues in ATP-bound PI3Kɣ are Ser 2165, Gln 2343, Asp 2357 , and Lys 2187. Amoung them Asp2357 and Lys 2187 were within 4 Å from the ligand.

2) Yang H, Rudge DG, Koos JD, et al. (2013) mTOR kinase structure, mechanism and regulation. Nature 497:217–223. doi: 10.1038/nature12122
